# Neuroimaging of sport concussion: persistent alterations in brain structure and function at medical clearance

**DOI:** 10.1038/s41598-017-07742-3

**Published:** 2017-08-24

**Authors:** Nathan W. Churchill, Michael G. Hutchison, Doug Richards, General Leung, Simon J. Graham, Tom A. Schweizer

**Affiliations:** 1 Neuroscience Research Program, St. Michael’s Hospital, Toronto, ON Canada; 2 Keenan Research Centre for Biomedical Science of St. Michael’s Hospital, Toronto, ON Canada; 3 Faculty of Kinesiology and Physical Education, University of Toronto, Toronto, ON Canada; 4 Department of Medical Imaging, University of Toronto, Toronto, Ontario Canada; 5 Department of Medical Biophysics, University of Toronto, Sunnybrook Hospital, Toronto, ON Canada; 6 Physical Sciences Platform, Sunnybrook Research Institute, Sunnybrook Health Sciences Centre, Toronto, ON Canada; 7 Faculty of Medicine (Neurosurgery) University of Toronto, Toronto, ON Canada

## Abstract

The medical decision of return to play (RTP) after a sport concussion is largely based on symptom status following a graded exercise protocol. However, it is currently unknown how objective markers of brain structure and function relate to clinical recovery. The goal of this study was to determine whether differences in brain structure and function at acute injury remain present at RTP. In this longitudinal study, 54 active varsity athletes were scanned using magnetic resonance imaging (MRI), including 27 with recent concussion, imaged at both acute injury and medical clearance, along with 27 matched controls. Diffusion tensor imaging was used to measure fractional anisotropy (FA) and mean diffusivity (MD) of white matter and resting-state functional MRI was used to measure global functional connectivity (Gconn). At acute injury, concussed athletes had reduced FA and increased MD, along with elevated Gconn; these effects remained present at RTP. Athletes who took longer to reach RTP also showed elevated Gconn in dorsal brain regions, but no significant white matter effects. This study presents the first evidence of altered brain structure and function at the time of medical clearance to RTP, with greater changes in brain function for athletes with a longer recovery time.

## Introduction

Concussion in sport and recreation is a growing health concern, with an estimated 1.6 to 3.8 million injuries occurring each year in the United States alone^1^. It is defined as biomechanical injury leading to altered brain function and its sequelae include somatic, cognitive and emotional disturbances, which are usually most severe within the first week post-injury^2^. Nonetheless, concussion is rarely associated with structural abnormalities on standard clinical neuroimaging, which may include computed tomography (CT) and magnetic resonance imaging (MRI)^3^. The clinical determination of safe return-to-play (RTP) is primarily based on self-reported symptoms, with medical clearance granted once the athlete is asymptomatic following a progressive exercise protocol^2^. Despite clinical recovery, there is evidence that individuals with a history of concussion are at risk for future injury^4, 5^ and in the long-term, they are at greater risk for depression and cognitive impairment^2, 6^. However, research on the determination of RTP has been largely epidemiological in nature, with limited information about the neurobiological changes that are associated with clinical recovery.

Advanced MRI is a promising tool for characterizing the subtle alterations in brain structure and function associated with concussion and recovery from injury. In this domain, Diffusion Tensor Imaging (DTI) is one of the most widely used MRI techniques, able to detect changes in the microstructure of brain tissue, based on properties of water diffusion. Typically, DTI is used to measure fractional anisotropy (FA; reflecting directionality of water diffusion) and mean diffusivity (MD; quantifying total water diffusion, independent of direction) in white matter, where water in myelinated fibers exhibits highly restricted, anisotropic diffusion. Most DTI studies of sport concussion have been cross-sectional, reporting altered FA and MD at acute injury (i.e., within the first week after sustaining a concussion)^7, 8^, along with significant white matter alterations months to years post-injury^9–11^ relative to uninjured controls. To date, only a few studies have examined longitudinal changes in white matter among concussed athletes, with Murugavel *et al*.^12^ showing recovery of microstructural abnormalities from 2 days to 2 weeks post-injury, whereas Henry *et al*.^13^ found no significant changes from acute concussion to 6 months post-injury.

Functional MRI (fMRI) provides a method for assessing brain function, based on regional fluctuations in blood oxygenation levels. Although less widely employed in concussion research than DTI, fMRI has proven to be highly sensitive to altered brain function following mild traumatic injury^14^. In particular, there has been growing interest in resting-state fMRI, where functional connectivity can be measured by correlating the fMRI time-series between different brain regions. This quantifies functional integration in the brain, which may be disrupted by the effects of disease and neurological insult^15, 16^. As with DTI, most resting-state fMRI studies of concussion have been cross-sectional, showing altered functional connectivity at acute injury^7^ and during the sub-acute interval from 1 week to 1 month post-concussion^17–19^, relative to uninjured controls. A longitudinal study by Zhu *et al*.^19^ reported significant reductions in functional connectivity from 1 to 7 days post-injury; however at present, little is known about functional recovery beyond the early phase of injury.

These findings have provided insight into the pathophysiology of concussion and preliminary evidence for MRI markers of brain recovery. Nevertheless, our understanding of concussion recovery remains at the very early stages. The few longitudinal MRI studies have focused on fixed post-injury time points, with no examination of neuroimaging markers specifically at the time of medical clearance. Moreover, these studies have generally focused on a single MRI modality, limiting our ability to compare functional and structural brain changes associated with recovery. The present study addresses these gaps, by acquiring DTI and fMRI scans for a group of concussed athletes (1) during the first week post-injury and (2) following medical clearance to RTP, along with a group of individually-matched control athletes. In addition, the changes in MRI measures from acute injury to RTP were regressed against recovery time, to test for specific markers of brain structure and function that are associated with a prolonged recovery. This study was conducted on a balanced sample of male and female athletes and a mixture of contact and non-contact sports, to identify MRI markers of concussion recovery that are broadly relevant to the sporting community.

## Results

### Demographics and clinical data

In this study, fifty-four (54) athletes were recruited from interuniversity (“varsity”) teams at a single institution. Twenty-seven (27) athletes were recruited following a physician diagnosis of concussion, and scanned at two time-points: the acute phase post-injury (1–7 days post-injury) and at medical clearance to return to play (RTP). For comparison, 27 individually-matched control athletes were also scanned, who had no concussions in the 6 months prior to imaging. Based on structural imaging, no abnormalities (e.g., clinically significant white matter hyper-intensities, contusions and micro-hemorrhage) were identified for the concussed athlete group in this study.

Table 1 summarizes athlete demographics, including symptom and cognitive scores for the Sport Concussion Assessment Tool 3 (SCAT3), at each assessment time-point. For athletes with concussion, time to RTP was highly variable, ranging from 4 days to approximately 8 months. At acute injury, concussed athletes had significantly higher total symptoms and symptom severity compared to their pre-season baseline assessments (mean ± standard error for total symptoms: 5.2 ± 1.4; *p* = 0.006; total severity: 13.4 ± 5.0; *p* = 0.012, based on non-parametric paired Wilcoxon tests). Acutely concussed athletes also have elevated symptoms compared to matched control athletes (total symptoms: 5.5 ± 1.3; *p* = 0.001; total severity: 12.8 ± 4.4; *p* = 0.004). At RTP, total symptoms and total severity were significantly reduced relative to acute injury (total symptoms: −7.6 ± 1.4; *p* < 0.001; total severity: −16.2 ± 4.7; *p* < 0.001), and also significantly lower than their pre-season baseline (total symptoms: −3.2 ± 0.9; *p* = 0.005; total severity: −4.7 ± 1.5; *p* = 0.001). All symptom effects remained significant after adjusting for multiple comparisons at a False-Discovery Rate (FDR) of 0.05. None of the other clinical measures were significantly different between acute injury and baseline, with *p* > 0.361 for all tests and concussed athletes showing median scores near or at the maximum value for most tests. This indicates that for SCAT3 clinical assessments of cognition, memory and balance, concussed athletes tended to perform at ceiling.

**Table 1:** Demographic data for athletes with concussion and matched controls, along with sport concussion assessment tool 3 (SCAT3) scores.

	Control	Concussion		
Age (mean ± SD)	20.1 ± 2.0	20.0 ± 1.8		
Female	14/27	14/27		
Number of previous concussions	0 [0,3]	1 [0,4]		
Days to RTP	—	18 [4,236]		
Sport	lacrosse (2) football (3) soccer (4) ice hockey (2) field hockey (4) volleyball (10) rugby (2)	water polo (1) lacrosse (3) basketball (3) rugby (9) football (3) ice hockey (2) field hockey (1) volleyball (4) soccer (1)		
		**Baseline**	**Acute**	**RTP**
Total Symptoms	2 [0,9]	2 [0,13]	5 [0,22]**	0 [0,4]
Symptom Severity	2 [0,11]	3 [0,24]	7 [0,90]**	0 [0,6]
Orientation	5 [4,5]	5 [4,5]	5 [4,5]	5 [4,5]
Immediate Memory	15 [10,15]	15 [13,15]	15 [13,15]	15 [13,15]
Concentration	3 [1,5]	4 [2,5]	4 [2,5]	4 [2,5]
Delayed Memory	4 [1,5]	5 [0,5]	4 [2,5]	5 [2,5]
Balance Total Errors	2 [0,10]	3 [0,11]	2 [0,9]	1 [0,9]

The SCAT3 scores are represented as the median [min, max]. ‘**’ Indicates a significant difference in scores for the acute concussion (“Acute”) time-point, relative to all other groups. Only Total Symptoms and Symptom Severity were significantly elevated at acute injury, relative to Baseline and matched controls.

### Neuroimaging data: from acute injury to RTP

White matter microstructure was evaluated using DTI to measure fractional anisotropy (FA) and mean diffusivity (MD) within white matter tracts. Resting brain function was evaluated using fMRI to measure global functional connectivity (Gconn), which quantifies total integrative brain function. To determine how MRI measures in concussed athletes evolved from acute injury to RTP, relative to the control group, Partial Least Squares (PLS) analysis was performed on each MRI measure (FA, MD, Gconn), to identify multivariate brain patterns (“voxel saliences”) that show greatest covariation between groups, with non-parametric bootstrap resampling used to assess significance of effects.

**Figure 1:**
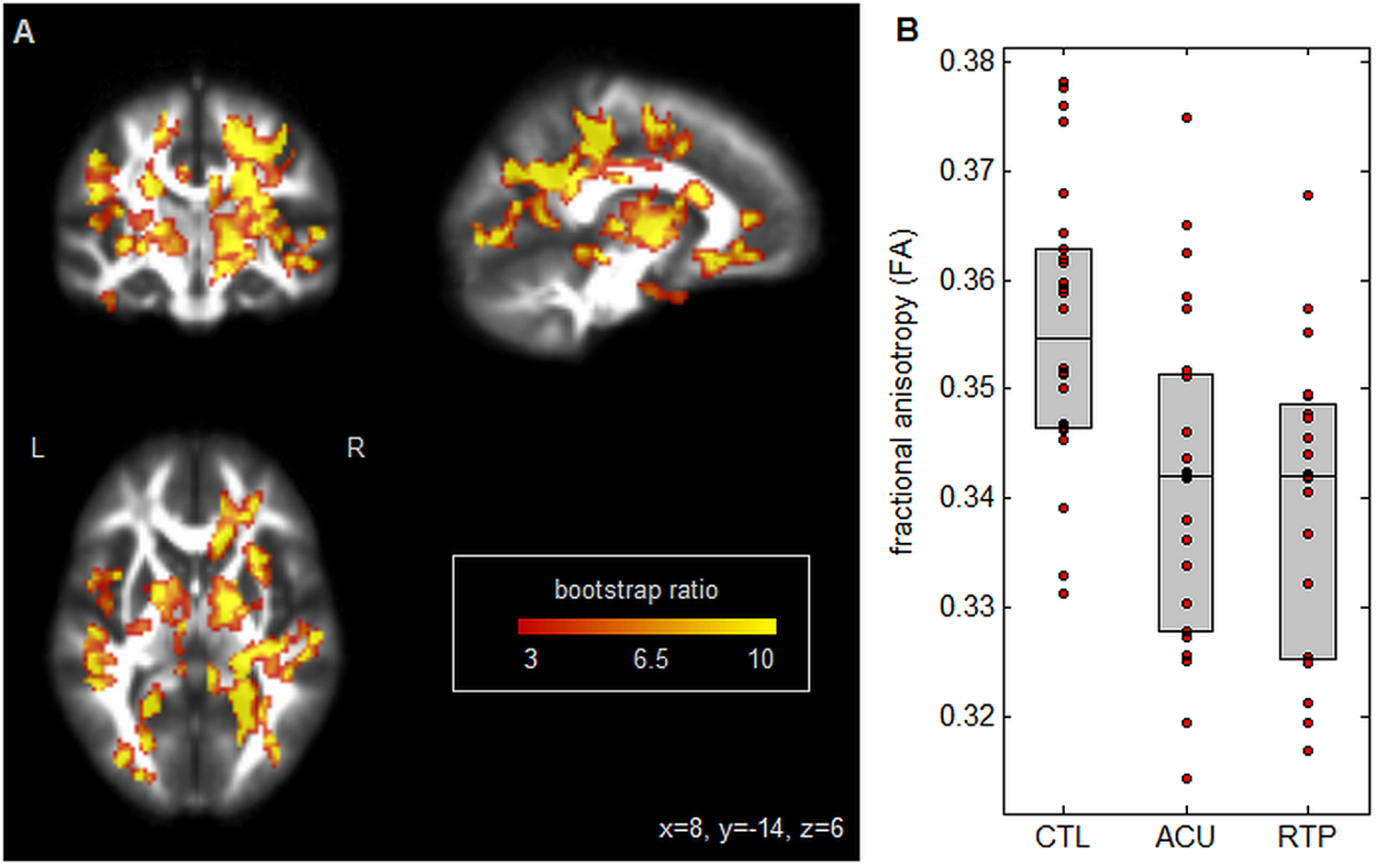
(**A**) Brain regions where fractional anisotropy (FA) shows significant differences between groups; effect sizes are reported as bootstrap ratios. Image shows maximum intensity projection (MIP) in each imaging plane, centered on the MNI coordinates (x = 8, y = −14, z = 6). (**B**) Average FA in significant brain regions, for control athletes (CTL) and for athletes with concussion imaged at acute injury (ACU) and at return-to-play (RTP). The boxes enclose upper and lower distribution quartiles, and middle line indicates the median. These results show reduced FA at both acute injury and RTP relative to controls, in the identified white matter regions.

Figure 1 shows PLS results for fractional anisotropy (FA). Widespread effects are seen in white matter tracts (Fig. 1A), including clusters located predominantly within the right corona radiata and bilaterally in posterior limbs of the internal capsule. As depicted in Fig. 1B, these regions showed reduced average FA relative to controls, for athletes at acute concussion (mean difference ± standard error: −0.0156 ± 0.003; *p* < 0.001, bootstrapped test of difference in PLS group saliences) and at RTP (mean difference: −0.0167 ± 0.0031; *p* < 0.001). The average FA had further decreased from acute injury to RTP, however, the effect was non-significant (mean change: −0.0013 ± 0.0020; *p* = 0.68), indicating persistent alterations in FA at medical clearance.

**Figure 2:**
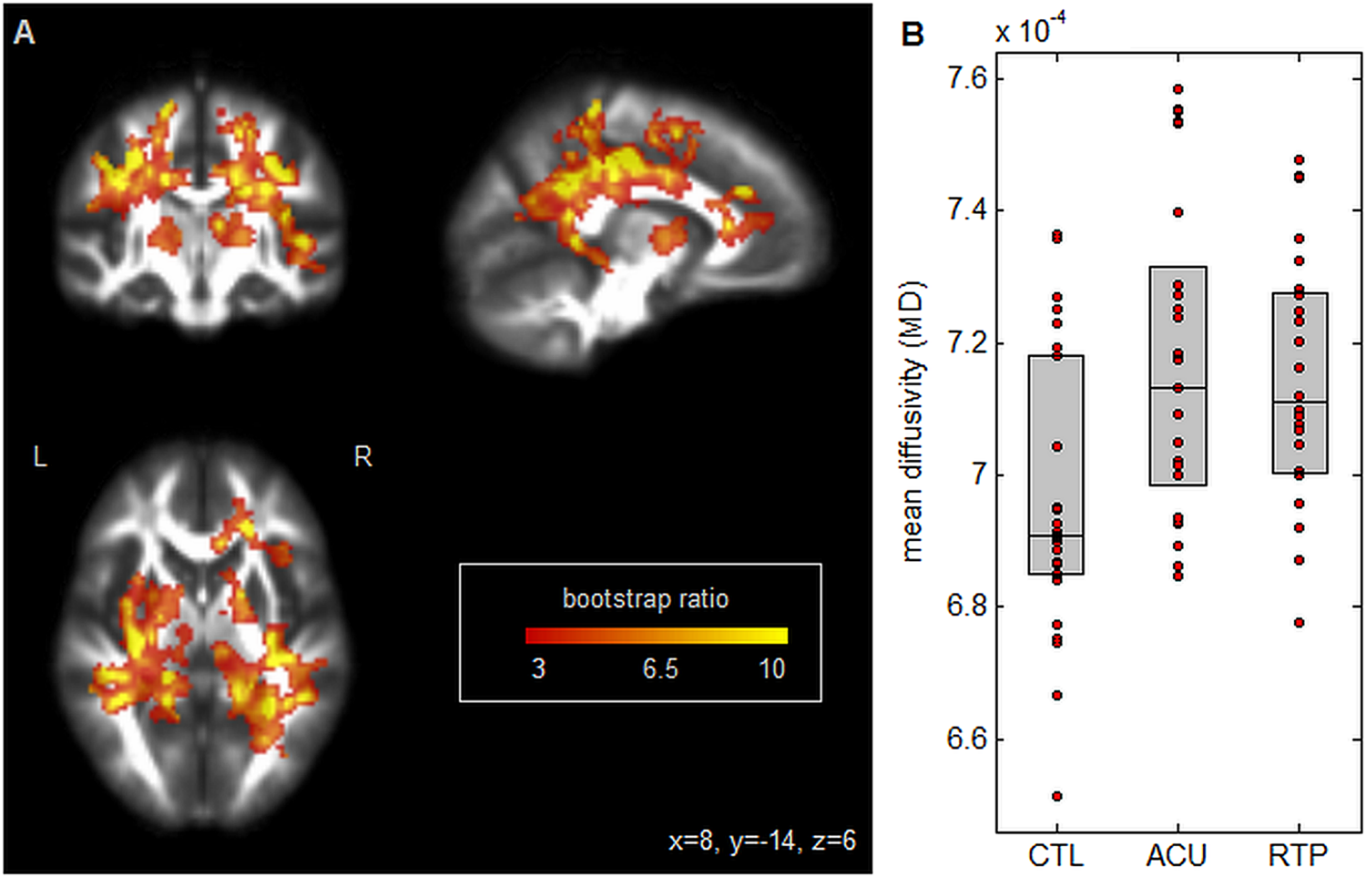
(**A**) Brain regions where mean diffusivity (MD) shows significant differences between groups; effect sizes are reported as bootstrap ratios. Image shows maximum intensity projection (MIP) in each imaging plane, centered on the MNI coordinates (x = 8, y = −14, z = 6). (**B**) Average FA in significant brain regions, for control athletes (CTL) and for athletes with concussion imaged at acute injury (ACU) and at return-to-play (RTP). The boxes enclose upper and lower distribution quartiles, and middle line indicates the median. These results show elevated MD at both acute injury and RTP relative to controls, in the identified white matter regions.

Figure 2 depicts PLS results for mean diffusivity (MD). Similar to FA, widespread effects are seen in white matter tracts (Fig. 2A), with clusters predominantly within bilateral corona radiata and posterior limbs of the internal capsule. As seen in Fig. 2B, these regions had increasing average MD relative to controls, at acute concussion (mean difference: (1.84 ± 0.66) × 10^−5^ mm/s; *p* = 0.004) and RTP (mean difference: (1.67 ± 0.52) × 10^−5^ mm/s; *p* < 0.001). Although average MD had decreased from acute injury to RTP, the effect was nonsignificant (mean change: (−0.11 ± 0.37) × 10^−5^ mm/s; *p* = 0.78), indicating persistent alterations in MD of white matter at the time of RTP.

**Figure 3:**
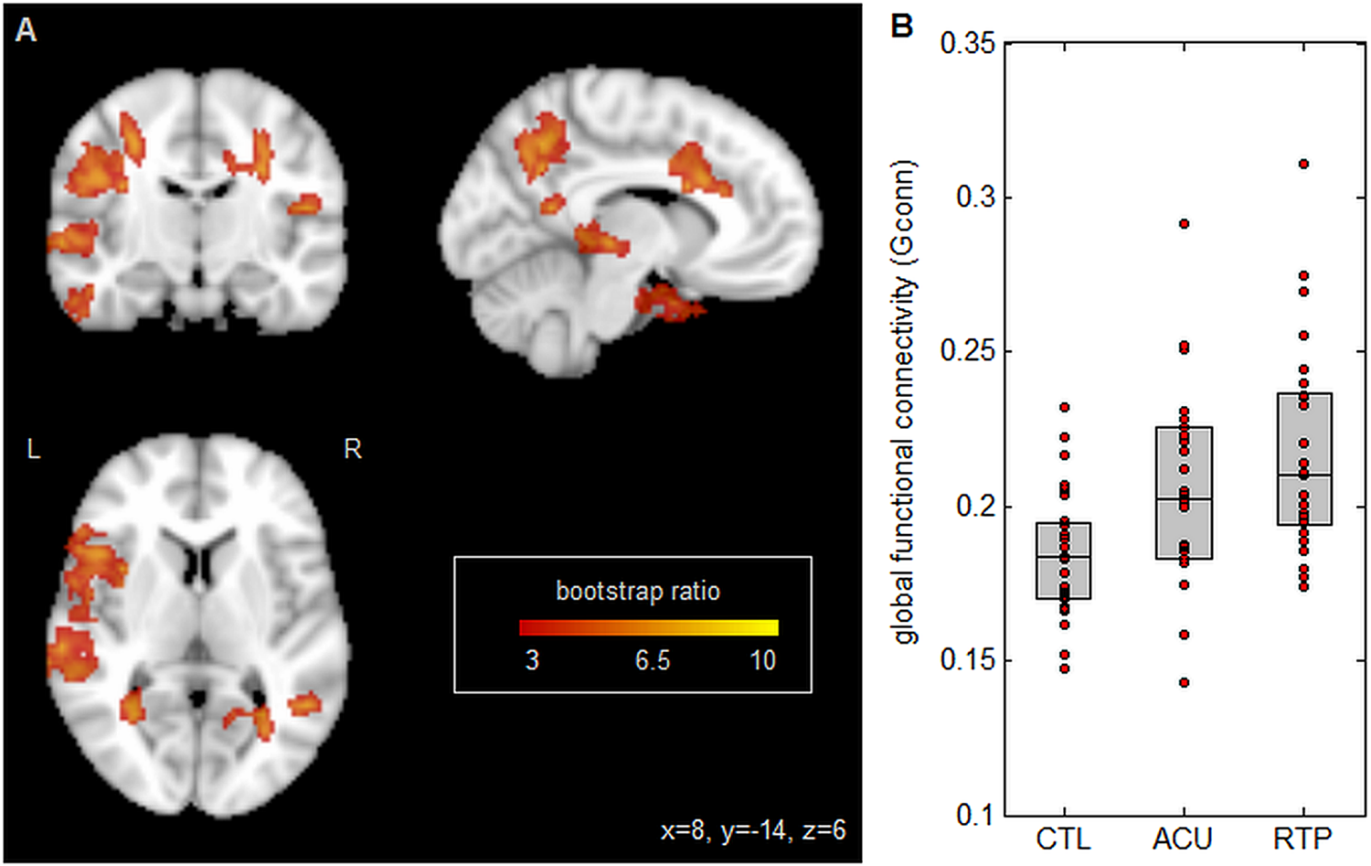
(**A**) Brain regions where global functional connectivity (Gconn) shows significant differences between groups; effect sizes are reported as bootstrap ratios. Image shows maximum intensity projection (MIP) in each imaging plane, centered on the MNI coordinates (x = 8, y = −14, z = 6). (**B**) Average Gconn in significant brain regions, for control athletes (CTL) and for athletes with concussion imaged at acute injury (ACU) and at return-to-play (RTP). The boxes enclose upper and lower distribution quartiles, and middle line indicates the median. These results show elevated Gconn at both acute injury and RTP relative to controls, in the identified grey matter regions.

Figure 3 shows PLS results for brain function, measured using global functional connectivity (Gconn). As with DTI, the most extensive changes are observed dorsally (Fig. 3A) in bilateral inferior parietal lobes and right angular gyrus, along with bilateral middle temporal gyri and the left inferior frontal lobe. As shown in Fig. 3B, average Gconn of athletes with acute concussion is significantly higher than controls (mean difference: 0.0310 ± 0.0120; *p* = 0.03), and continues to increase relative to controls at RTP (mean difference: 0.0325 ± 0.0073; *p* < 0.001). However, the difference between acute injury and RTP was non-significant (mean change: 0.002 ± 0.008; *p* = 0.92), indicating persistently altered resting brain function at the time of medical clearance.

### Neuroimaging data: effects of recovery time

**Figure 4:**
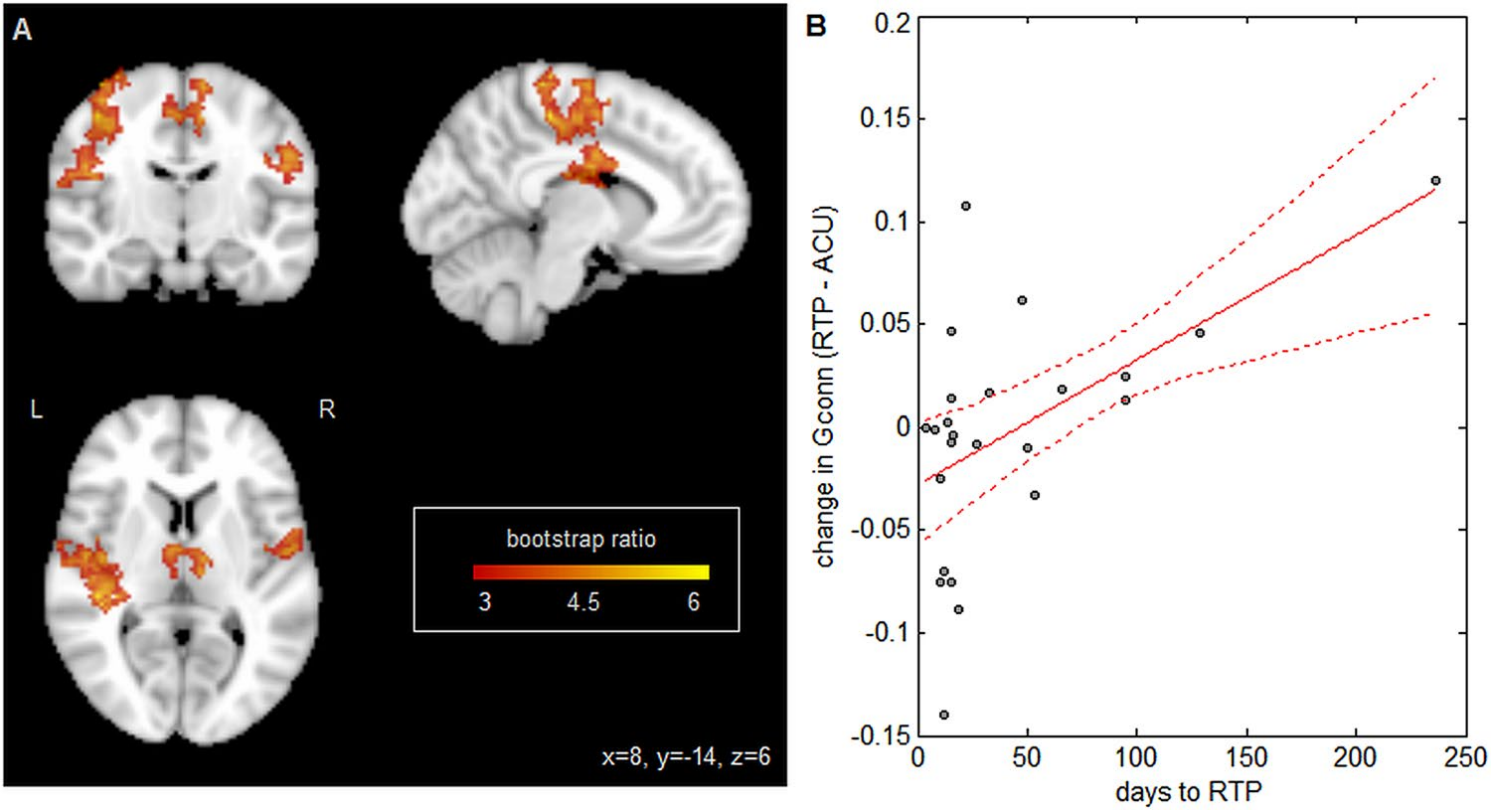
Relationship between global functional connectivity (Gconn) and days to RTP. (**A**) Brain regions where Gconn is reliably predicted by days to RTP. Effect sizes are reported as bootstrap ratios (mean/standard error). Image shows maximum intensity projection (MIP) in each imaging plane, centered on the MNI coordinates (x = 8, y = −14, z = 6). (**B**) Individual subject Gconn changes in significant brain regions, plotted against days to RTP. The line of best fit is plotted in solid red, with 95% confidence bounds given by dashed lines. These results show greater elevations in Gconn at RTP, for athletes with a longer recovery time.

An additional set of analyses tested whether there are patterns of brain change from acute injury to RTP that are associated with prolonged clinical recovery. For each MRI measure (FA, MD, Gconn) the set of within-subject paired differences in brain maps (RTP – acute) was regressed against total number of days from concussion to RTP, with bootstrapping used to assess significance of effects. Neither of the DTI measures showed significant association between brain changes at RTP (relative to acute injury) and the number of days to RTP, after adjusting for multiple comparisons. However, changes in Gconn were significantly related to time to RTP (Fig. 4A), within the supplementary motor area, paracentral lobule and middle cingulum, along with left precentral and bilateral postcentral gyri. Figure 4B plots mean Gconn of significant brain voxels against days to RTP, with a regression coefficient of determination *R* ^2^ = 0.282 (95% confidence interval: 0.027, 0.631). As seen in the plot, athletes with shorter recovery times (i.e., less than one month) tended to have decreased Gconn at RTP relative to acute injury, whereas athletes with longer recovery times tended to have increased Gconn. Therefore, while concussed athletes exhibit regions of elevated connectivity that are unaltered from acute injury to RTP (Fig. 3), a separate set of brain regions show significant connectivity changes from acute injury to RTP, with the direction of effect depending on the time interval of clinical recovery.

## Discussion

This study used advanced MRI to evaluate concussed athletes at both acute injury and medical clearance to RTP, to better understand the neurobiological changes related to clinical recovery. This addresses a significant gap in the literature, as prior longitudinal studies have largely focused on fixed post-injury time intervals. In addition, the investigation was conducted on a balanced sample of male and female athletes and a mixture of contact and non-contact sports, which reinforces the general relevance of these findings to the sporting community. MRI measures of brain structure and function were analyzed in a flexible multivariate PLS framework, to detect spatially distributed changes in the brains of concussed athletes. The primary findings of this study included persistent alterations in white matter microstructure and resting brain function at medical clearance to RTP, potentially indicating ongoing biological processes in the brain that persist beyond clinical recovery.

The DTI analyses revealed significantly altered brain microstructure at both acute injury and RTP, including decreased FA and elevated MD, which are consistent with prior studies of early injury^9, 12^. These effects may be driven by multiple physiological responses to concussion. Acute brain injury has been linked to intracellular edema, where the loss of ionic homeostasis leads to cellular swelling, with glial cells being predominantly affected^20, 21^. However, for some cohorts, intracellular edema has been associated with compressed interstitial spaces and elevated FA^8^. Brain injury may also induce vasogenic edema, where disruption of the blood-brain barrier causes fluid uptake within interstitial spaces^20^. The present results are consistent with glial-mediated intracellular edema and vasogenic edema, both of which may reduce FA and increase MD. Another potential contributor is neuroinflammation due to disruptions of the blood-brain barrier, which may be caused by head impacts during sport participation^22^. Glial activation is a key component of the neuroinflammatory response^23^, thereby reducing FA and increasing MD. Other candidates for the observed microstructural changes include axonal injury, demyelination and structural reorganization^24–26^, although they are typically associated with more severe brain injury and longer post-injury time intervals. At present, we have an incomplete understanding of how these different processes contribute to the observed microstructural changes following a concussion, making this an important area of future investigation.

For both FA and MD, no significant differences were seen between acute injury and RTP, and changes in these measures were not significantly associated with days to RTP. This suggests that there are limited changes in brain microstructure within the examined post-concussion time interval (i.e., a median of 18 days from injury to RTP, ranging from 4 days to 8 months). These findings are supported by a prior DTI meta-analysis, which identified consistent long-term white matter abnormalities associated with mild traumatic brain injury^27^. In the sport domain, previous studies have reported altered white matter for athletes with a history of concussion, scanned months to years post-injury^10, 11, 13^. However, these studies typically found elevated FA and reduced MD, which is opposite to the present findings, suggesting that different neurobiological processes may predominate at the time of medical clearance, compared to long-term effects associated with a history of concussion.

The resting-state fMRI analyses showed elevated Gconn for concussed athletes relative to matched controls, at both acute injury and RTP. These findings are aligned with a prior resting-state fMRI study of the long-term effects of concussion^28^, which found elevated functional connectivity among athletes with a greater number of prior concussions, including the parietal and inferior frontal regions identified in this paper. The increased functional connectivity seen at both acute injury and RTP has been previously observed in various forms of neurological insult, including stroke and more severe brain injury^29^, which suggests that this may be a consistent functional brain response to neural injury. Elevated functional connectivity may a consequence of neurometabolic dysfunction, which occurs after a concussive impact^30^. Alternatively, it has been proposed that elevated functional integration reflects a greater redundancy in brain function and may serve as a protective mechanism following traumatic injury^29^. The present findings may therefore reflect an adaptive response to injury, which would help to maintain function and mitigate negative outcomes if a second injury is acquired during recovery from the initial concussion.

Altered brain function in dorsal brain regions has been previously reported for a range of traumatic brain injury severities^27, 29^. The parietal and angular gyri identified in Fig. 3 are critical for visual and sensory integration, while the inferior frontal lobe plays a role in cognitive control, including task switching and response inhibition^31, 32^. In addition, the middle temporal gyri showed altered connectivity for concussed athletes; this region is implicated in semantic functions, including the processing of action knowledge^33^. Dysfunction in these regions is especially concerning, as deficits in visual integration and response are common sequelae of concussion^2^ and the proper functioning of these domains is critical for athletes to avoid re-injury during active sport participation. This is particularly important, as there is literature evidence of more severe consequences associated with a second concussion, if it occurs within a fixed time-window after the initial injury^34^.

The localization of both DTI and fMRI changes to the dorsal part of the brain provide greater support for our findings and suggests a common (or strongly linked) process of brain change. However, the correlation of changes in Gconn with time to RTP indicates that this marker of brain function is more strongly linked to clinical recovery than the DTI measures, with longer recovery time and more persistent symptoms being correlates of hyper-connectivity. Interestingly, the association with days to RTP was primarily observed in regions implicated in sensorimotor function, indicating that altered connectivity in these regions may be a specific marker of prolonged recovery for athletes with concussion.

Although the current findings provide strong evidence of ongoing brain recovery at RTP, there are a few issues which should be addressed in future work. Athletes post-injury were compared to a sample of matched controls, however, they would ideally be compared to their un-injured baseline MRI scan. Nonetheless, the current protocol of individually matching controls to concussed athletes provides a relatively strong comparison. The persistence of differences in brain structure and function at RTP also raises the question of when these differences are expected to dissipate. Thus, it will be crucial for future studies to examine within-subject changes in the months to years following symptom resolution, to determine if, and when, functional and structural markers of concussion have dissipated. In addition, there is growing literature evidence showing significant sex differences in concussion incidence and clinical outcomes^35, 36^. While this study shows robust neuroimaging findings for combined male and female athlete groups, future research should also examine whether there is a neuroimaging basis for the observed sex differences in concussion outcome.

The present work reports the first evidence of ongoing brain changes in athletes with concussion at the time of clinical recovery. These findings significantly extend our understanding of the pathophysiology of concussion, and help to motivate future work investigating brain changes relative to the time of symptom resolution. Ultimately, pursuing this avenue of neuroimaging research may lead to refinements in concussion management strategies to minimize the potential risk of re-injury for athletes recovering from concussion.

## Materials and Methods

### Study participants

Fifty-four (54) athletes were recruited from interuniversity teams at a single institution (including volleyball, hockey, soccer, football, rugby, basketball and lacrosse). Twenty-seven (27) athletes were recruited following a physician diagnosis of concussion. Formal physician evaluation for suspected concussion was aligned with the standardized framework set forth by the Concussion in Sport Group^2^, for events where athletes sustained direct or indirect contact to head with the presence of signs and/or symptoms. Concussed athletes were scanned at two time-points: the acute phase post-injury (1–7 days post-injury) and following medical clearance to return to play (RTP). Acute imaging was conducted a median of 4 days post-injury, while RTP imaging was conducted a median of 7 days post-clearance. Medical clearance to RTP was determined by normal Sport Concussion Assessment Tool 3 (SCAT3) scores, along with successful completion of both a graded exercise protocol consistent with consensus guidelines^2^ and a computerized cognitive screening battery. Demographic and clinical information were also collected, including prior concussion history. For comparison, 27 control athletes were also imaged who had no concussions within the preceding 6 months. Controls were exactly matched on sex and presence of multiple prior concussions, and showed no significant difference in age (mean difference: 0.15 ± 1.03; *p* = 0.59, paired Wilcoxon test). Two athletes with concussion were not imaged at RTP due to drop-out, while one athlete was scanned at RTP but did not have acute imaging, leaving 24 athletes with complete acute and RTP scans. The study procedures were approved by research ethics boards (REBs) at the University of Toronto and St. Michael’s Hospital, carried out in accordance with REB guidelines, and all patients provided written informed consent prior to study participation.

### Magnetic Resonance Imaging

Participants were imaged at St. Michael’s Hospital, using an MRI system operating at 3 Tesla (Magnetom Skyra, Siemens, Erlangen, Germany) with the standard 20-channel head receiver coil. Anatomical imaging included a T1-weighted Magnetization Prepared Rapid Acquisition Gradient Echo (MPRAGE) sequence. Participants were also imaged with structural sequences including fluid attenuated inversion recovery imaging (FLAIR) and susceptibility-weighted imaging (SWI) to identify any structural abnormalities, including lesions and micro-hemorrhage. DTI and fMRI sequences were acquired afterwards. During fMRI acquisition, participants were instructed to lie still with their eyes closed, and not focus on anything in particular. The eyes closed condition was employed in this group particularly to avoid confounds or compliance issues associated acute concussion, such as photosensitivity and post-concussion headache.

#### Structural Imaging

T1-weighted MPRAGE was obtained, with field-of-view (FOV) = 24 × 24 cm, 240 × 240 × 192 matrix, 0.9 mm isotropic voxels, bandwidth (BW) = 250 Hz/Pixel, inversion time (TI)/echo time (TE)/repetition time (TR) = 850/2.63/2000 ms, flip angle (FA) = 8°. FLAIR was obtained with FOV = 22 × 18.6 cm, 256 × 196 matrix, 1.1 × 0.9 × 3.0 mm voxels, BW = 315 Hz/Pixel, TI/TE/TR = 2200/96/9000 ms. SWI was also obtained, with 220 × 192 FOV, 0.6 × 0.6 × 1.2 mm voxels. TR/TE 28/20 ms, FA = 15°, 0.2 mm encoding gap, BW = 120 Hz/px).

#### White Matter Microstructure

Diffusion-weighted imaging was based on 30-directions encoding at b = 700 s/mm^2^, FOV = 24 × 24 cm, 120 × 120 acquisition matrix, 66 axial slices, 2 mm isotropic voxels, bandwidth = 1736 Hz/Pixel, TE/TR = 23/7800 ms. The FSL *eddy_correct* protocol was used to perform simultaneous correction of eddy currents and rigid-body motion correction, FSL *bet* was used to mask out non-brain voxels, and FSL *dtifit* was used to calculate voxel-wise fractional anisotropy (FA) and diffusivity measures. Co-registration of Diffusion-weighted brain maps was based on the FSL FDT protocol, described as follows: (1) masked subject FA maps were eroded by 1 voxel width at brain edges, and co-registered to the FMRIB58 template (1 mm^3^) via affine transform, using FSL *flirt*. Afterwards, (2) a symmetric, study-specific template was computed by averaging transformed subject FA maps, then re-averaging with flipped left/right orientations. This was repeated with (3) the average template as a reference and non-linear registration of FA maps using FSL *fnirt*, which was then used to update the study-specific template. This step was repeated once more, by (4) performing nonlinear registration and updating the mean template. Prior to analysis, all images were convolved with a 6 mm FWHM 3D Gaussian kernel to minimize the effects of local variation in white matter structure. All analyses were performed within a mask of regions with mean FA > 0.25, to focus on effects primarily within white matter tracts.

#### Brain Function

Resting-state fMRI data were obtained via multi-slice T2*-weighted echo planar imaging (FOV = 20 × 20 cm, 64 × 64 matrix, 32 slices, 3.125 × 3.125 × 4.5 mm voxels, BW = 2232 Hz/Pixel, TE/TR = 30/2000, FA = 70°, oblique axial interleaved) to produce a time-series of 193 samples images. Subsequent data processing and analysis were performed using software from the Analysis of Functional Neuroimages (AFNI) package (afni.nimh.nih.gov) and customized algorithms developed in the laboratory. This included rigid-body motion correction (AFNI *3dvolreg*), removal of outlier scan volumes (using nitrc.org/projects/spikecor), slice-timing correction (AFNI *3dTshift*), spatial smoothing with a 6 mm Full Width at Half Maximum (FWHM) isotropic 3D Gaussian kernel (AFNI *3dmerge*), regression of motion parameters and linear-quadratic trends as nuisance covariates. To control for physiological noise due to heartbeat and respiration, data-driven physiological correction was performed (via nitrc.org/projects/phycaa_plus), along with regression of white matter signal, by segmenting the brain with the FSL *fast* algorithm and regressing out the mean signal in white matter voxels with *P* > 0.95. Co-registration of fMRI data was obtained by computing the rigid-body transform of the time averaged fMRI data for each participant to their T1-weighted anatomical image, and the 12-parameter affine transformation of the anatomical image for each participant to the MNI152 template. The transformation matrices were concatenated and the net transform applied to all fMRI data, resampled at 2 × 2 × 2 mm^3^ resolution.

### Demographics and clinical data

The SCAT3 scores were compared at each post-concussion time-point, relative to both baseline test scores and the test scores of the matched controls, using non-parametric paired Wilcoxon tests. Uncorrected p-values were reported, along with significant differences after correcting for multiple comparisons at a False Discovery Rate (FDR) of 0.05.

### Neuroimaging data: from acute injury to RTP

Multivariate Partial Least Squares (PLS) analysis was performed for each of the MRI measures (FA, MD, Gconn), to identify patterns of significant covariation between groups^37–39^. For each MRI measure, a mean-centered task PLS analysis was performed on the mean brain patterns for (1) matched controls and athletes with concussion at (2) acute injury and (3) RTP. Each PLS analysis produced paired components, including a “voxel salience” map reflecting brain regions that showed greatest covariation across groups, and a set of “group saliences”, reflecting how much each group expressed this brain pattern. The first PLS component is reported for each of the analyzed MRI measures, which explains the greatest total covariance across groups.

Repeated-measures bootstrap resampling was used to generate empirical distributions on the PLS component (1000 iterations), with resampling units consisting of a subject’s acute and RTP scans, along with their matched control. The effect size for voxel saliences was expressed as the bootstrap ratio of each variable (mean/standard error), corrected for multiple comparisons by applying a voxel-level threshold at *p* = 0.005, followed by cluster-size thresholding using Analysis of Functional Neuroimages (AFNI; afni.nimh.nih.gov/afni) program *3dFWHMx* to estimate spatial smoothness and *3dClustSim* to identify the minimum cluster size at an adjusted *α* = 0.05 significance. All brain maps are shown as a set of maximum intensity projections (MIPs) in each imaging plane, centered on the MNI coordinates (x = 8, y = −14, z = 6). In addition, the mean MRI measure (FA, MD or Gconn) was computed over significant brain voxels for each subject, and the distribution of these values plotted for each group. To ascertain which groups showed significant differences in expression of MRI measures, bootstrapped p-values were computed on the difference in group saliences between each pair of sport groups, with significant differences reported after adjusting at a FDR of 0.05.

### Neuroimaging data: effects of recovery time

An additional analysis was conducted to determine whether changes in the brain are correlated with the time interval from injury to medical clearance. For each MRI measure, this was done by computing the within-subject paired difference in MRI values (RTP – acute) at each voxel, and performing ordinary least-squares regression against the total number of days from concussion to RTP. For consistency with the PLS analysis described above, results were evaluated via bootstrap resampling on subjects (1000 iterations), and the significance of voxel-wise regression coefficients was evaluated based on the bootstrap ratio. The same multiple comparison correlation was applied as above, by adjusting to a cluster-wise significant of *α* = 0.05.

## References

[1] Langlois JA, Rutland-Brown W, Wald MM (2006). The epidemiology and impact of traumatic brain injury: a brief overview. J Head Trauma Rehabil..

[2] McCrory P (2013). Consensus statement on concussion in sport: the 4th International Conference on Concussion in Sport held in Zurich, November 2012. British Journal of Sports Medicine.

[3] Yuh EL, Hawryluk GW, Manley GT (2014). Imaging concussion: a review. Neurosurgery.

[4] Guskiewicz K (2003). Cumulative effects associated with recurrent concussion in collegiate football players: the NCAA Concussion Study. JAMA: the Journal of American Medical Association.

[5] Abrahams S, Mc Fie S, Patricios J, Posthumus M, September AV (2014). Risk factors for sports concussion: an evidence-based systematic review. British journal of sports medicine.

[6] Guskiewicz K (2007). Recurrent concussion and risk of depression in retired professional football players. Med.Sci.Sports Exerc.

[7] Churchill NW (2017). The first week after concussion: blood flow, brain function and white matter microstructure. NeuroImage: Clinical.

[8] Wilde E (2008). Diffusion tensor imaging of acute mild traumatic brain injury in adolescents. Neurology.

[9] Cubon V, Putukian M, Boyer C, Dettwiler A (2011). A diffusion tensor imaging study on the white matter skeleton in individuals with sports-related concussion. Journal of neurotrauma.

[10] Sasaki T (2014). Hockey Concussion Education Project, Part 3. White matter microstructure in ice hockey players with a history of concussion: a diffusion tensor imaging study: Clinical article. Journal of neurosurgery.

[11] Churchill, N. *et al*. Brain structure and function associated with a history of sport concussion: a multi-modal MRI study. *Journal of neurotrauma* (in press) (2016). 10.1089/neu.2016.4531 27246317

[12] Murugavel M (2014). A Longitudinal Diffusion Tensor Imaging Study Assessing White Matter Fiber Tracts after Sports-Related Concussion. Journal of neurotrauma.

[13] Henry LC (2011). Acute and chronic changes in diffusivity measures after sports concussion. Journal of neurotrauma.

[14] Slobounov S, Gay M, Johnson B, Zhang K (2012). Concussion in athletics: ongoing clinical and brain imaging research controversies. Brain imaging and behavior.

[15] Van Den Heuvel MP, Pol HEH (2010). Exploring the brain network: a review on resting-state fMRI functional connectivity. European neuropsychopharmacology.

[16] Grefkes C, Fink GR (2014). Connectivity-based approaches in stroke and recovery of function. The Lancet Neurology.

[17] Johnson B (2012). Alteration of brain default network in subacute phase of injury in concussed individuals: resting-state fMRI study. Neuroimage.

[18] Zhang K (2012). Default mode network in concussed individuals in response to the YMCA physical stress test. Journal of neurotrauma.

[19] Zhu D (2015). A Potential Biomarker in Sports-Related Concussion: Brain Functional Connectivity Alteration of the Default-Mode Network Measured with Longitudinal Resting-State fMRI over Thirty Days. Journal of neurotrauma.

[20] Unterberg A, Stover J, Kress B, Kiening K (2004). Edema and brain trauma. Neuroscience.

[21] Marmarou A (2007). A review of progress in understanding the pathophysiology and treatment of brain edema. Neurosurgical focus.

[22] Marchi N (2013). Consequences of repeated blood-brain barrier disruption in football players. PloS one.

[23] Streit WJ, Mrak RE, Griffin WST (2004). Microglia and neuroinflammation: a pathological perspective. Journal of neuroinflammation.

[24] Sidaros A (2008). Diffusion tensor imaging during recovery from severe traumatic brain injury and relation to clinical outcome: a longitudinal study. Brain.

[25] Kraus MF (2007). White matter integrity and cognition in chronic traumatic brain injury: a diffusion tensor imaging study. Brain.

[26] Arfanakis K (2002). Diffusion tensor MR imaging in diffuse axonal injury. American Journal of Neuroradiology.

[27] Eierud C (2014). Neuroimaging after mild traumatic brain injury: review and meta-analysis. NeuroImage: Clinical.

[28] Churchill, N. *et al*. Changes in functional connectivity of the resting brain associated with a history of sport concussion. *Brain Injury* (in press) (2016). 10.1080/02699052.2016.1221135 27901587

[29] Hillary F (2015). Hyperconnectivity is a fundamental response to neurological disruption. Neuropsychology.

[30] Giza C, Hovda D (2001). The neurometabolic cascade of concussion. Journal of athletic training.

[31] Aron AR, Robbins TW, Poldrack RA (2004). Inhibition and the right inferior frontal cortex. Trends in cognitive sciences.

[32] Derrfuss J, Brass M, Neumann J, von Cramon DY (2005). Involvement of the inferior frontal junction in cognitive control: Meta‐analyses of switching and Stroop studies. Human brain mapping.

[33] Binder JR, Desai RH, Graves WW, Conant LL (2009). Where is the semantic system? A critical review and meta-analysis of 120 functional neuroimaging studies. Cerebral Cortex.

[34] Vagnozzi R (2008). Temporal Window Of Metabolic Brain Vulnerability To Concussion: A Pilot 1 h‐Magnetic Resonance Spectroscopic Study In Concussed Athletes—Part III. Neurosurgery.

[35] Dick R (2009). Is there a gender difference in concussion incidence and outcomes?. British Journal of Sports Medicine.

[36] Preiss-Farzanegan S, Chapman B, Wong T, Wu J, Bazarian J (2009). The relationship between gender and postconcussion symptoms after sport-related mild traumatic brain injury. PM&R.

[37] Krishnan A, Williams L, McIntosh A, Abdi H (2011). Partial Least Squares (PLS) methods for neuroimaging: a tutorial and review. Neuroimage.

[38] McIntosh A, Chau W, Protzner A (2004). Spatiotemporal analysis of event-related fMRI data using partial least squares. Neuroimage.

[39] Rosipal, R. & Krämer, N. In *Subspace, latent structure and feature selection* 34–51 (Springer, 2006).

